# Critical role of the mast cell/tryptase/PAR2 axis in anastrozole-induced pain

**DOI:** 10.1111/bph.70280

**Published:** 2025-12-14

**Authors:** Maria Fernanda Pessano Fialho, Raquel Tonello, Evelyne Silva Brum, Gabriela Becker, Nigel W. Bunnett, Sara Marchesan Oliveira

**Affiliations:** 1 Graduate Program in Biological Sciences: Toxicological Biochemistry, Centre of Natural and Exact Sciences, Federal University of Santa Maria, Santa Maria, Brazil; 2 Department of Molecular Pathobiology, Pain Research Center, New York University, New York, New York, USA; 3 Graduate Program in Biological Sciences: Biochemistry, Department of Biochemistry, Institute of Basic Health Sciences, Universidade Federal do Rio Grande do Sul, Porto Alegre, Brazil

**Keywords:** aromatase inhibitors, G protein-coupled receptors, mastocytosis, musculoskeletal pain

## Abstract

**Background and Purpose:**

Anastrozole, an aromatase inhibitor, is used to treat postmenopausal women with hormone receptor-positive breast cancer, but also induces musculoskeletal pain and can lead to therapeutic regimen suspension. Aromatase inhibitors promote the release of pro-inflammatory substances from sensitised nerve fibres, which might lead to peripheral mast cell activation.

**Experimental Approach:**

We explore if tryptase released by peripheral mast cells could activate the protease-activated receptor 2 (PAR2) to sustain anastrozole-induced painful symptoms.

**Key Results:**

Anastrozole caused mechanical allodynia, muscle strength loss, and increased mast cell number and tryptase levels in the plantar tissue of male C57BL/6 mice. Depletion (using compound 48/80) or stabilisation (using ketotifen fumarate) of mast cells prevented anastrozole-induced mechanical allodynia and muscle strength loss. Compound 48/80 also prevented the increase in the number of mast cells in the plantar tissue. Tryptase inhibitors nafamostat and gabexate, or PAR2 inhibitors ENMD-1068 and AZ3451, reduced the anastrozole-induced mechanical allodynia and muscle strength loss. Furthermore, anastrozole did not cause mechanical allodynia and muscle strength loss in global (PAR2^−/−^) or sensory neuron-specific (PAR2 Na_v_ 1.8^−/−^) PAR2 knockout mice. Local sub-nociceptive doses of the PAR2 agonists (tryptase, trypsin or 2F) enhanced the anastrozole-induced mechanical sensitivity in wild-type mice, which was reduced by pre-treatment with AZ3451. PAR2^−/−^ or PAR2 Na_v_ 1.8^−/−^ mice treated with anastrozole did not respond to local sub-nociceptive doses of PAR2 agonists.

**Conclusions and Implications:**

Our results provide a new mechanism underlying anastrozole-induced pain, highlighting the mast cell/tryptase/PAR2 axis as a therapeutic target to manage painful symptoms.

## 1 | INTRODUCTION

Cancer is a major global health issue and a leading cause of morbidity and mortality (Siegel et al., 2025; Sung et al., 2021). Cancer incidence has risen in recent decades because of increased life expectancy, lifestyle changes and improved diagnostic methods. Breast cancer is the most common form of cancer among women worldwide, with an estimated 2.3 million new cases annually (Bray et al., 2024; Siegel et al., 2023). A valuable therapeutic approach for women diagnosed with hormone receptor-positive breast cancer is adjuvant endocrine therapy with aromatase inhibitors that contributes to improving the quality of life and survival rates of patients (Tenti et al., 2020; Waks & Winer, 2019).

Aromatase inhibitors are recommended for postmenopausal women for a duration of 5 to 10 years following a breast cancer diagnosis, with anastrozole being the most commonly prescribed aromatase inhibitor (64% of cases) (Tenti et al., 2020; Waks & Winer, 2019). Although this drug class is considered safe and promising, many patients report significant adverse effects from treatment onset (Henry et al., 2008; Laroche et al., 2014; Waks & Winer, 2019). Painful musculoskeletal symptoms, characterised by morning stiffness, joint pain, myalgia, and decreased strength, are common daily complaints among aromatase inhibitor users (Henry et al., 2008; Tenti et al., 2020). The persistence of daily painful symptoms combined with the ineffectiveness of analgesic drugs significantly affects the quality of life and treatment compliance, leading to at least a quarter of patients discontinuing therapy (Burstein et al., 2019; Henry et al., 2008). Therefore, understanding the underlying mechanisms of these painful conditions is essential in the search for new therapies to attenuate the pain associated with the use of aromatase inhibitors.

Efforts have been made to elucidate the mechanisms underlying painful symptoms associated with aromatase inhibitor treatment (Fusi et al., 2014; De Logu et al., 2016; Robarge et al., 2016; Brusco et al., 2023; Fialho et al., 2023, 2024). Current studies have focused on the involvement of G protein-coupled receptors and ion channels expressed on sensory neurons. Their activation facilitates sensory neuron sensitisation, contributing to painful symptoms (Fusi et al., 2014; Brusco et al., 2023; Fialho et al., 2023, 2024). Once sensory neurons are sensitised, they release pro-inflammatory substances (Fusi et al., 2014) that potentially promote several cellular responses, such as peripheral mast cell activation (Baral et al., 2019; Chatterjea & Martinov, 2015; Gupta & Harvima, 2018).

Mast cells are located near nociceptor terminals in peripheral tissues (Baral et al., 2019; Chatterjea & Martinov, 2015; Gupta & Harvima, 2018). Upon activation, mast cells release tryptase, which cleaves the protease-activated receptor 2 (PAR2) on neuronal and non-neuronal cells (Baral et al., 2019; Chatterjea & Martinov, 2015; Héron & Dubayle, 2013; Peach et al., 2023). Tryptase activates PAR2, a G protein-coupled receptor, through canonical mechanisms (Gupta & Harvima, 2018; Molino et al., 1997; Peach et al., 2023; Vergnolle, Wallace, et al., 2001). Activation of PAR2 triggers intracellular signalling pathways and cellular responses that lead to painful symptoms and inflammatory responses (Peach et al., 2023; Vergnolle, Bunnett, et al., 2001), including those strongly related to symptoms caused by aromatase inhibitors (Cheng et al., 2011; Kume et al., 2023; Lucena & McDougall, 2020; Muley et al., 2016; Sakamoto et al., 2016).

Here, we have tested the hypothesis that aromatase inhibitors promote the release of pro-inflammatory factors from sensitised nerve terminals, triggering the recruitment and activation of mast cells in peripheral tissue. Mast cell mobilisation leads to tryptase release, activating PAR2 expressed on sensory neurons, which contributes to the painful symptoms associated with anastrozole use. Our results, in a mouse model, demonstrated that tryptase from peripheral mast cells activated PAR2 on sensory neurons, thereby sustaining the painful symptoms induced by anastrozole.

## 2 | METHODS

### 2.1 | Preparation of drugs

Anastrozole was dissolved in 0.5% carboxymethylcellulose (CMC). Ketotifen fumarate (oral solution, 1 mg ml^−1^) was acquired from Biosintetica^®^ (São Paulo, Brazil). Gabexate (tryptase inhibitor) and ENMD-1068 (PAR2 antagonist) were dissolved in 5% DMSO (final concentration administered). Trypsin and tryptase (PAR2 agonists) were prepared as stock solutions in phosphate-buffered saline (PBS; 10 mM). 2-Furoyl-LIGRLO-amide (2F; PAR2 agonist) and AZ3451 (PAR2 negative allosteric modulator; McIntosh et al., 2020) were prepared as stock solutions in 100% DMSO. All the stock solutions were diluted in PBS to the desired concentration just before use. The final concentration of DMSO in all drug solutions did not exceed 5%; 5% DMSO alone did not cause any detectable effects per se in the mice. The control groups (indicated as vehicle) received only the vehicles in which the treatments were dissolved. Oral, intraperitoneal and subcutaneous treatments were administered in mice in a volume of 10 ml kg^−1^. The volume of the intraplantar (i.pl.) injection did not exceed 20 μl per paw. The doses of the drugs used in this study were based on previous studies (Jaffrey et al., 1994; Oliveira et al., 2011, Oliveira et al., 2013; Fialho et al., 2023, 2024; da Silva Brum et al., 2024).

### 2.2 | Animals

All animal care and experimental protocols were approved by the Institutional Animal Care and Use Committee from the Federal University of Santa Maria (protocol #9467111022) or New York University (NYU; protocol #201900130). Animal studies are reported in compliance with the ARRIVE guidelines (Percie du Sert et al., 2020) and with the recommendations made by the *British Journal of Pharmacology* (Lilley et al., 2020).

Adult C57BL/6 mice (male, 20–25 g, 7–8 weeks) were produced and provided by the Central Animal Facility from the Federal University of Santa Maria. C57BL/6 wild-type (PAR2^+/+^), and PAR2-globally deficient (PAR2^−/−^) mice were from Jackson Laboratories (Bar Harbor, ME, USA). PAR2-deficient sensory neuron-specific (PAR2 Na_v_ 1.8^−/−^) mice (20–25 g, 8–10 weeks) were generated by heterozygous mice on a C57BL/6 background. These mice were produced and provided by New York University. The animals were kept under controlled environmental conditions with 12:12 h light–dark cycle, ambient temperature (22 ± 1°C) and relative humidity of 55 ± 10%. The animals were fed a standard pellet diet and allowed free access to water. Mice were humanely killed with an overdose of sodium thiopental (150 mg kg^−1^), given intraperitoneally (i.p.), followed by cervical dislocation to ensure death. Some groups of mice were killed by asphyxiation with inhaled CO_2_ plus 10–50% O_2_, and death was confirmed by cervical dislocation. The choice of male mice was based on previous studies (Fusi et al., 2014; Brusco et al., 2023; Fialho et al., 2023, 2024) and their low oestrogen levels, similar to postmenopausal women undergoing breast cancer treatment with anastrozole (Tenti et al., 2020; Waks & Winer, 2019). Furthermore, the choice of male mice was based on the findings of Li et al. (2025), who demonstrated that aged, naturally menopausal, female mice and young male mice exhibited comparable mechanical pain sensitivity, as well as similar anxiety-like and depressive-like behaviours following aromatase inhibitor treatment.

Behavioural studies were conducted following the ARRIVE guidelines (Percie du Sert et al., 2020) and the recommendations provided by the *British Journal of Pharmacology* (Lilley et al., 2020). The behavioural studies followed the national and international legislation (guidelines of the Brazilian Council of Animal Experimentation and the U.S. Public Health Service’s Policy on Humane Care and Use of Laboratory Animals). The group size of six animals used for each in vivo experiment was based on studies that used protocols similar to ours (Brum et al., 2020; Fischer et al., 2020; Brusco et al., 2023; Fialho et al., 2023, 2024). A randomisation procedure (http://www.randomizer.org/) was used to ensure allocation concealment, as per the baseline thresholds, prior to treatment administration. The intensities of noxious stimuli were the minimum necessary to demonstrate the consistent effects of the treatments. Experimental procedures were conducted in accordance with the current guidelines for laboratory animal care and the ethical guidelines for investigations of experimental pain in conscious animals established by the International Association for the Study of Pain (Kilkenny et al., 2010).

### 2.3 | Anastrozole-induced pain model

To induce painful behaviours in mice, we used anastrozole at a dose of 0.2 mg kg^−1^, based on the human dose of 1 mg recommended by the Food and Drug Administration to treat breast cancer-diagnosed postmenopausal women (Gibson et al., 2009). We used the conversion factor from human to mouse indicated by the National Institute of Health (Reagan-Shaw et al., 2008) and according to previous studies (Zameer & Vohora, 2017; Brusco et al., 2023; Fialho et al., 2023, 2024). The experimental protocol consisted of administering anastrozole (0.2 mg kg^−1^) by oral route (p.o.) or vehicle (control group, 10 ml kg^−1^, p.o.; 0.5% CMC). As single or repeated (for 15 days) treatment with anastrozole caused consistent and similar painful behaviours in mice on all evaluated days (Fialho et al., 2023), we opted for a single administration of anastrozole to investigate the contribution of mast cells and PAR2 to this model of anastrozole-induced pain.

#### 2.3.1 | Study design for behavioural assessment

Baseline measurements for paw withdrawal threshold (PWT) (assessed using von Frey filaments) and muscle strength (assessed using the Grip test or Kondziela inverted screen test) were taken before administration (baseline, B) and again at 2- or 3-h post-administration of either anastrozole or vehicle (time 0, baseline 2). Subsequently, animals underwent various treatment protocols (see more details below), and the painful behavioural parameters were assessed at different times, following the treatments. Additionally, we selected the time frame (in hours) in which i.pl. treatment with agonists produced the most significant nociceptive effect in anastrozole-treated mice to gauge the pharmacological effects of tryptase inhibitors and PAR2 antagonists.

#### 2.3.2 | Protocols

##### Assessment of mast cell involvement

Firstly, we evaluated whether the development of painful behaviours (mechanical allodynia and muscle strength loss) after anastrozole treatment might be associated with an increase in mast cell number (mastocytosis), as assessed by histological analysis of the plantar tissue at 3 h after the administration of anastrozole or vehicle. The experimental design is represented in Figure 1a. The non-evoked pain-related behaviours were measured 3 h after the anastrozole or vehicle treatment (the experimental design is represented in Figure S1A).

To mitigate severe allergic and inflammatory reactions (Karadag et al., 2000), we administered compound 48/80, which depletes mast cells (Jaffrey et al., 1994), to mice at escalating doses (0.5 mg kg^−1^ on the first day, 1 mg kg^−1^ on the second day, 2 mg kg^−1^ on the third day and 4 mg kg^−1^ on the fourth day) by the i.p. route twice daily. Following mast cell depletion, the animals underwent the protocol of anastrozole-induced pain. The effect of compound 48/80 on the mechanical PWT and muscle strength loss was assessed from 0.5 to 7 h after anastrozole administration. The histological parameters following the compound 48/80 protocol were assessed 3 h after the administration of either anastrozole or vehicle. The experimental design is represented in Figure 2a.

Ketotifen (10 mg kg^−1^), a mast cell membrane stabiliser, was orally administered to mice once daily for five consecutive days. On the sixth day, the animals received either anastrozole or vehicle administration. The effect of ketotifen on mechanical PWT and muscle strength loss was assessed from 2 up to 7 h after anastrozole administration. The experimental design is represented in Figure 3a.

##### Contribution of tryptase

Firstly, the protease levels (including tryptase) of the plantar tissue were analysed 3 h after the administration of anastrozole or vehicle. Next, to evaluate the contribution of tryptase enzyme on mechanical allodynia and muscle strength loss induced by anastrozole, mice were treated with a single administration of nafamostat (10 mg kg^−1^, p.o.) or gabexate (10 mg kg^−1^, subcutaneous, s.c.), both tryptase inhibitors, or their vehicles (10 ml kg^−1^, p.o. or s.c.) at 2 h after anastrozole administration. The mechanical PWT (from 0.5 to 3 h) and muscle strength (only at 1 h) were measured after the tryptase inhibitors treatment, as represented in Figure 4a.

##### Contribution of PAR2

To assess the involvement of PAR2 on painful symptoms induced by anastrozole, mice received a single administration of ENMD-1068 (10 mg kg^−1^, i.p., PAR2 antagonist) or AZ3451 (60 μg kg^−1^, i.p., PAR2 negative allosteric modulator), or their vehicles (10 ml kg^−1^, i.p.) at 2 h after anastrozole administration. The mechanical PWT (from 0.5 to 3 h) and muscle strength loss (only at 1 h) were measured after the PAR2 inhibitor treatments. The experimental design is represented in Figure 5a.

We employed two genetic approaches to confirm the participation of PAR2 in painful symptoms induced by anastrozole: mice with global deletion (PAR2 −/−) or sensory neuron-specific knockout (PAR2 Na_v_ 1.8^−/−^) of PAR2 (Jimenez-Vargas et al., 2018; Latorre et al., 2022). Both mouse strains underwent the protocol of anastrozole-induced pain. The mechanical PWT (from 1 to 6 h) and muscle strength (only at 3 h) were assessed after anastrozole administration. The experimental design is represented in Figure 6a.

We also investigated whether sub-nociceptive doses of PAR2 agonists could enhance the painful behaviours of the anastrozole-treated mice. For this, the animals were previously treated with anastrozole or vehicle. After 2 h, the animals received i. pl. injections of tryptase (3 ng/paw), trypsin (3 ng/paw), or 2F (100 ng/paw) or their vehicles (20 μl/paw). Three other groups of animals received AZ3451 at 2 h after anastrozole. After 0.25 h of AZ3451 treatment, the same animals were treated with sub-nociceptive doses of PAR2 agonists, tryptase, trypsin or 2F by the i.pl. route. After 1 h of agonist injections (at the time of maximum nociception induced by the agonists), the mechanical PWT was measured to evaluate the antinociceptive effect of the PAR2 negative allosteric modulator AZ3451. The experimental design is represented in Figure 7a.

In another subset of experiments, we treated PAR2^−/−^ or PAR2 Na_v_ 1.8^−/−^ mice with anastrozole, and after 2 h, the animals received i.pl. injections of tryptase (3 ng/paw), trypsin (3 n/paw), or 2F (100 ng/ paw) or their vehicles (20 μl/paw). The mechanical PWT was assessed from 0.5 to 3 h after anastrozole administration. The experimental design is presented in Figure S2A.

### 2.4 | Behavioural experiments

#### 2.4.1 | Assessment of mechanical PWT

The assessment of PWT, using the von Frey test, is widely used in both preclinical and clinical settings to evaluate mechanical allodynia (Kostek et al., 2016; Oliveira et al., 2013). The assessment of mechanical PWT was carried out using flexible nylon filaments (von Frey) of increasing stiffness (0.02–10 g) by the Up-and-Down method (Chaplan et al., 1994; Oliveira et al., 2013). The 50% PWT to the mechanical stimulus was calculated from the resulting scores as previously described (Chaplan et al., 1994; Dixon, 1980). The mechanical PWT was expressed in grams (g), and a significant decrease in the PWT compared with the baseline values or vehicle groups was considered as mechanical allodynia.

#### 2.4.2 | Assessment of muscle strength

The loss of muscle strength/muscle weakness test in mice was evaluated using a grip strength meter (Grip Strength meter EFF 305 – Insight, Ribeirão Preto, SP, Brazil) (Brum et al., 2020; Fialho et al., 2023, 2024), a test used in both preclinical and clinical settings to assess loss of muscle strength/muscle weakness, which is considered an indicator of musculoskeletal pain (Lintermans et al., 2011; Montilla-García et al., 2017). The test consists of the experimenter gently holding the mouse by the base of the tail, allowing the animal to grab the metal bar with the forelimbs and hindlimbs before being gently pulled until it releases its grip. The forelimb and hindlimb muscle strength, expressed in grams (g), was measured 3 times per mouse with at least 1-min interval between measurements. A significant decrease in muscle strength compared to the baseline or vehicle groups was considered a loss of muscle strength/muscle weakness, indicative of musculoskeletal pain.

Loss of muscle strength was also evaluated in a subset of mice using the inverted screen test, Kondziela test, another test to assess all limbs’ loss of muscle strength/muscle weakness in animals (Deacon, 2013). Thus, mice were placed in the centre of the wire mesh screen, and the apparatus was inverted. The time elapsed before the mouse fell from the screen was recorded (s). The 1-min cut-off time was used (Dagnino et al., 2019).

#### 2.4.3 | Non-evoked pain-related behaviours

The non-evoked pain-related behaviours in mice, a validated and unbiased tool for rodent phenotyping that minimises operator influence (Brodkin et al., 2014), were evaluated using a behavioural spectrometer from Behavioural Instruments. This apparatus comprises a 40-cm^2^ arena featuring a charge-coupled device (CCD) camera positioned in the centre of the ceiling and a door aperture in the front area of the arena. Movement was tracked through a floor-mounted vibration sensor and 32 wall-mounted infrared transmitter and receiver pairs. Mice were placed individually within the spectrometer’s centre, observed for 20 min, and their behaviours were recorded. Analysis was conducted using video tracking methods (Viewer3, BiObserve, Sankt Augustin, Germany) and vibration analysis. Total activity travelled in the open field (% activity), the average velocity of locomotion (centimetres per second), track length (centimetres), ambulation (% activity) and time engaged in grooming (min) were recorded and analysed (Brodkin et al., 2014; Tonello et al., 2023).

### 2.5 | Ex vivo *or* in vitro experiments

#### 2.5.1 | Histological analysis of mast cells

To evaluate the infiltration and degranulation of mast cells in the plantar tissue of the anastrozole-treated mice, we conducted histological analyses. Samples were collected 3 h after the vehicle or anastrozole treatment. Mice were killed, and their plantar tissues were collected and immersed in 10% neutral buffered formalin. Each sample was embedded in paraffin, sectioned at 5 μm, and stained with 0.2% toluidine blue. Histological slides were analysed using an optical microscope (Axio Scope.A1, Carl Zeiss). Histological images were captured at 200X, 400X or 1000X total magnification (20X, 40X or 100X objectives, with a 10 × ocular lens) using a camera (Axiocam 105 colour). To quantify the number of mast cells and their degranulation, we counted two fields from six different histological slides (images at 200X magnification) of each experimental group using ImageJ software (NIH, USA). When the classification of degranulated mast cells was uncertain, additional images were carefully examined at higher magnification (400X, using a 40X objective with a 10X ocular lens) to ensure accuracy. Mast cell degranulation was considered when granules showed staining alterations and/or were located outside the cell boundary. The results were expressed as the number of mast cells per field or the number of degranulated mast cells per field.

#### 2.5.2 | Measurement of protease activity

Protease levels (including tryptase) were assessed using the colorimetric assay kit (ab102531; Abcam) according to the manufacturer’s instructions. Briefly, plantar tissue from vehicle or anastrozole-treated mice was homogenised in assay buffer and centrifuged at 13,000 × *g* for 10 min at 4°C. The total protein concentration of the supernatant was measured by bicinchoninic acid (BCA) analysis, which was then used to measure protease levels. Protease levels are expressed as protease nanomoles (nmol) per milligram protein, compared to its standard curve.

#### 2.5.3 | Calcium mobilisation assay

Human kidney epithelial cells (HEK293, #CRL-3216^™^, American Type Culture Collection), which express endogenous PAR2 (hPAR2-HEK293) were cultured in Dulbecco’s modified Eagle’s medium (DMEM) supplemented with foetal bovine serum (FBS, 10%), L-glutamine (2 mM), penicillin (100 U ml^−1^) and streptomycin (100 mg ml^−1^). Cells were maintained at 37°C in 5% CO_2_ and 95% O_2_. hPAR2-HEK293 cells were incubated on PDL-coated 96-well plates (35,000 cells per well). Cells were loaded with fura-2 AM (10 μM, Cayman Chemicals) in loading buffer (150 mM NaCl, 2.6 mM KCl, 0.1 mM CaCl_2_, 1.18 mM MgCl_2_, 10 mM D-glucose, 10 mM HEPES, 4 mM probenecid, 0.5% BSA, pH 7.4) for 1 h at 37°C. After, the cells were washed and placed in assay buffer. hPAR2-HEK293 cells were challenged with vehicle (buffer control), trypsin (0.01–10 μM, final concentration), a PAR2 agonist, or anastrozole (30–300 μM, final concentration). Some experiments were performed in the presence of the PAR2 negative allosteric modulator AZ3451 (10 μM, final concentration) with a 15-min preincubation. Ionomycin (10 μM, final concentration), dissolved in DMSO and diluted with the buffer solution, was applied at the end of each experiment as a positive control for the assay. Changes in mobilisation of intracellular calcium were monitored by sequential dual excitation at 340 and 380 nm and emission at 510 nm and recorded with a dynamic image analysis system (FlexStation 3 Multi-Mode Microplate Reader). Results were expressed as the percentage increase in the ratio 340/380 above the baseline and normalised to the maximum effect induced by ionomycin (% change in R_340/380_) (Fusi et al., 2014).

### 2.6 | Data and statistical analyses

The data and statistical analysis adhere to the recommendations of the *British Journal of Pharmacology* for experimental design and analysis in pharmacology (Curtis et al., 2025). The results are presented as the mean + SEM. Parametric data were analysed using one-tailed Student’s *t* test or one- or two-way ANOVA, followed by Bonferroni’s post hoc test when appropriate, using GraphPad Prism 8.0 software (San Diego, CA, U.S.A.). Mechanical threshold data underwent a log transformation before analysis to meet parametric assumptions. Inhibition percentages were calculated based on the maximum developed responses compared to baseline 1 value or the control group. Post hoc tests were conducted only if the *F* in ANOVA achieved *P* < 0.05. Sample sizes subjected to statistical analysis were at least six mice per group (*n* = 6), where *n* = the number of independent values. *P* < 0.05 was considered to show a statistically significant difference between group means. Statistical tests and the sample size used for each analysis are shown in the figure legends.

### 2.7 | Materials

All reagents used in these experiments were analytical grade and, when not otherwise indicated, were purchased from Sigma-Aldrich Chemical Company (St. Louis, MO, USA). Anastrozole was obtained from Sun Farmacêutica do Brasil Ltda (Goiânia, Brazil). 2-Furoyl-LIGRLO-amide (2F) and AZ3451 were purchased from MedChemExpress (Monmouth Junction, NJ, USA), and nafamostat mesylate from Fisher Scientific (Waltham, MA, USA).

### 2.8 | Nomenclature of targets and ligands

Key protein targets and ligands in this paper are hyperlinked to corresponding entries at https://www.guidetopharmacology.org and are permanently archived in the Concise Guide to PHARMACOLOGY 2023/24 (Alexander, Christopoulos et al., 2023; Alexander, Fabbro et al., 2023).

## 3 | RESULTS

### 3.1 | Peripheral mast cells are involved in the anastrozole-induced painful symptoms

Anastrozole treatment markedly decreased PWT to mechanical stimulation, characterised as mechanical allodynia, (Figure 1b) and decreased muscle strength, characterised as muscle weakness (Figure 1c), compared to the vehicle group at 3 h after treatment. The number of mast cells increased 3.6-fold (Figure 1d and e), and mast cell degranulation increased threefold (Figure 1f) in the plantar tissue 3 h after anastrozole treatment, compared to the vehicle group), establishing a relation between mast cell infiltration and the development of anastrozole-associated painful symptoms. In contrast, anastrozole did not affect non-evoked pain-like behaviour at 3 h after its administration (Figure S1). Because anastrozole treatment did not cause non-evoked pain-related behaviour (spontaneous pain), we focused our subsequent experiments on stimulus-triggered pain responses after anastrozole treatment, specifically mechanical allodynia and muscle strength.

### 3.2 | Mast cell depletion and stabilisation prevented anastrozole-induced painful symptoms

Depletion of mast cells by repeated treatment with compound 48/80 (0.5, 1, 2 and 4 mg kg^−1^, i.p.) prevented the decrease in mechanical PWT and the loss of muscle strength at all times evaluated until the effect of anastrozole was no longer observed (Figure 2b and c), compared to vehicle plus anastrozole group. Compound 48/80 reduced the number of mast cells to baseline values (vehicle plus vehicle group) compared to the anastrozole group (Figure 2d and e).

Pre-treatment of mice with the mast cell membrane stabiliser ketotifen fumarate also prevented the decrease in mechanical PWT and muscle strength loss until the effects of anastrozole were no longer apparent (Figure 3b and c), compared with the vehicle plus anastrozole group. Taken together, our findings suggest that peripheral mast cells are involved in the painful symptoms associated with anastrozole.

### 3.3 | Tryptase contributes to anastrozole-induced painful symptoms

Firstly, we evaluated the protease activity in the plantar tissue of mice after anastrozole treatment. Protease levels were increased 1.6-fold in plantar tissue, compared with control levels (Figure 4b), at 3 h after anastrozole treatment. Mice were treated with two different tryptase inhibitors to confirm the involvement of tryptase in the painful symptoms induced by anastrozole. Nafamostat, a clinically used tryptase inhibitor, reversed the mechanical allodynia (Figure 4c) and muscle strength loss (Figure 4d) in the mice, from 0.5 to 2 h and at 1 h after its administration, respectively. Gabexate, another inhibitor of tryptase, also reversed the mechanical allodynia (Figure 4e) and muscle strength loss from 0.5 to 1 h and at 1 h after its administration in mice, respectively (Figure 4f).

### 3.4 | PAR2 mediates anastrozole-induced painful symptoms

#### 3.4.1 | PAR2 antagonism reduced the development of anastrozole-induced painful symptoms

Once we observed the increased protease levels in peripheral tissue and the tryptase contribution to the anastrozole-induced painful symptoms, we subsequently evaluated the involvement of PAR2 (a tryptase-sensitive receptor) in these symptoms. ENMD-1068, a PAR2 antagonist, reduced the mechanical allodynia from 0.5 to 2 h (Figure 5b) and muscle strength loss at 1 h (Figure 5c) after anastrozole treatment. The PAR2 negative allosteric modulator AZ3451 also reversed the mechanical allodynia from 0.5 to 1 h (Figure 5d) and muscle strength loss at 1 h (Figure 5e) after anastrozole treatment.

#### 3.4.2 | Deletion of PAR2 reduces anastrozole-induced painful symptoms

In wild-type C57BL/6 mice, anastrozole reduced mechanical PWT from 1 to 3 h (Figure 6b and d) and diminished muscle strength at 3 h (Figure 6c and e) after its administration, compared with baseline values. Depletion of PAR2 globally (PAR2^−/−^ global) or from sensory neurons (PAR2 Na_v_ 1.8^−/−^) attenuated the reduction of mechanical PWT (Figure 6b and d) and the loss of muscle strength (Figure 6c and e) induced by anastrozole, confirming a role for neuronal PAR2.

#### 3.4.3 | Effect of PAR2 agonists on mechanical allodynia in anastrozole-treated animals

The i.pl. injection of sub-nociceptive doses of the PAR2 agonists, tryptase (3 ng/paw), trypsin (3 ng/paw) or 2F (100 ng/paw), enhanced the mechanical sensitivity after 1 h in anastrozole-treated mice when compared to the anastrozole plus vehicle group (Figure 7b–d). The PAR2 negative allosteric modulator AZ3451 (administered 0.25 h before injection of the agonists) reduced the mechanical hypersensitivity caused by tryptase, trypsin and 2F at 1 h injected into the paw (Figure 7b–d). However, the injection of sub-nociceptive doses of PAR2 agonists did not worsen the loss of muscle strength in anastrozole-treated mice, compared with the anastrozole plus vehicle group (data not shown). As expected, the sub-nociceptive doses of the tryptase, trypsin or 2F, injected in the paw, did not alter the mechanical PWT and muscle strength loss in animals previously treated with vehicle (10 ml kg^−1^, p.o.) (data not shown). Furthermore, sub-nociceptive doses of tryptase, trypsin or 2F did not modify the mechanical PWT of PAR2^−/−^ or PAR2 Na_v_ 1.8^−/−^ mice previously treated with anastrozole, over the evaluated time (from 0.5 to 3 h) (Figure S2).

#### 3.4.4 | Anastrozole does not directly activate PAR2

To explore whether anastrozole directly activated human PAR2, we used HEK293 cells expressing endogenous human PAR2 (hPAR2–HEK293). In these cells, anastrozole alone did not evoke concentration-dependent calcium responses (Figure 8a). However, trypsin, a classical PAR2 agonist, did induce concentration-dependent calcium responses (Figure 8b), which were abolished in the presence of the PAR2 negative allosteric modulator AZ3451 (Figure 8b).

## 4 | DISCUSSION

Aromatase inhibitors often cause musculoskeletal pain, which can undermine the therapeutic regimen (Crew et al., 2007; Henry et al., 2008; Laroche et al., 2014). In this study, anastrozole caused evoked pain-related behaviours depending on the activation and recruitment of peripheral mast cells, followed by tryptase release and indirect activation of PAR2 on sensory neurons, which sustain painful symptoms. Nevertheless, anastrozole did not cause non-evoked pain-related behaviours (spontaneous pain) in mice, according to a previous study (Fusi et al., 2014). It is essential to be clear that aromatase inhibitors are used clinically in patients surviving breast cancer and not in healthy subjects, as in our model, which used healthy mice. However, given the clinical complexity of distinguishing pain arising from the tumour itself, surgical procedures or treatment with anti-neoplastic drugs (Bennett et al., 2019), it is essential to identify the mechanisms triggered by each factor, individually, to improve pain management. For this reason, we chose to investigate the effects of the aromatase inhibitor in isolation, without the presence of breast tumours or of any relevant surgical procedure.

While well-tolerated, approximately one-third of the patients treated with anastrozole experience musculoskeletal adverse effects, which can impair quality of life and clinical outcomes (Crew et al., 2007; Henry et al., 2008; Laroche et al., 2014; Waks & Winer, 2019). Although some studies have sought to clarify the feed-forward mechanisms of these painful symptoms, additional pro-algesic pathways that operate alone or together might also contribute to the pain associated with the use of aromatase inhibitors (Fusi et al., 2014; De Logu et al., 2016; Brusco et al., 2023; Fialho et al., 2023, 2024).

Mast cells are proinflammatory cells (Galli et al., 2005; Gupta & Harvima, 2018; Mittal et al., 2019) that modulate pain responses by several neuronal mechanisms (Baral et al., 2019; Chatterjea & Martinov, 2015; Gupta & Harvima, 2018). Once activated, the mast cells undergo degranulation, releasing algogenic molecules that act on receptors expressed on the surface of sensory fibres and the mast cells themselves, establishing bidirectional positive feedback. It leads to the continuous release of inflammatory mediators and neuronal sensitisation, contributing to painful perception (Baral et al., 2019; Gupta & Harvima, 2018; Mittal et al., 2019). Once aromatase inhibitors promote the release of neuropeptides from neuronal cells (Fusi et al., 2014), they may play a crucial role in activating and mobilising mast cells to peripheral tissues, thereby significantly contributing to the painful symptoms associated with anastrozole use.

Mast cell depletion or mast cell membrane stabilisation has been used in research to confirm the role of mast cells in painful disorders (Klooker et al., 2010; Meloto et al., 2021; Oliveira et al., 2011; Oliveira et al., 2013). Our results align with previous data, demonstrating that the depletion or membrane stabilisation of mast cells reduces painful parameters in pre-clinical pain models (Chatterjea et al., 2012; da Silva Brum et al., 2024; Meloto et al., 2021; Oliveira et al., 2011; Oliveira et al., 2013). Ketotifen fumarate is a safe drug approved by health agencies, and preliminary clinical evidence has demonstrated its analgesic effect (Klooker et al., 2010; Meloto et al., 2021). So, its repositioning for use concomitantly with aromatase inhibitors would be an attractive combination to be assessed clinically. In terms of our model, our data would support a significant contribution by peripheral mast cells to anastrozole-associated pain.

Mast cells release inflammatory substances mediating pathological processes (Baral et al., 2019; Gupta & Harvima, 2018), such as the protease tryptase (Gupta & Harvima, 2018; Molino et al., 1997). In some pre-clinical or clinical pain conditions, the increased levels of tryptase are considered a marker for mast cell activation, directly related to the painful symptoms (Aich et al., 2015; Borbély et al., 2016; Hoffmeister et al., 2011; Lam & Schmidt, 2010; Mai et al., 2021; Oliveira et al., 2013; Schwartz et al., 1987). Here, anastrozole increased the protease levels in the paw tissue of mice, which corroborates our peripheral mastocytosis results and the maximum nociception of anastrozole. Furthermore, gabexate and nafamostat transiently reversed anastrozole-induced mechanical allodynia and muscle strength loss in mice. Our results agree with previous studies demonstrating that tryptase inhibitors exert anti-nociceptive effects in pre-clinical pain models (da Silva Brum et al., 2024; Hoffmeister et al., 2011; Oliveira et al., 2013). Nafamostat has demonstrated a favourable safety profile in clinical use (Hernández-Mitre et al., 2022; Narumi et al., 2023) and may serve as a translational option for treating painful conditions, associated with elevated protease levels.

Activation of PAR2 by tryptase triggers painful symptoms or inflammatory responses (Peach et al., 2023; Vergnolle, Wallace, et al., 2001). The increased tryptase levels observed after anastrozole treatment and the contribution of PAR2 to several painful conditions (Peach et al., 2023) make this receptor a pivotal target for painful symptoms associated with aromatase inhibitor use. The PAR2 antagonist ENMD-1068 (Kelso et al., 2006) and the PAR2 negative allosteric modulator AZ3451 (Kennedy et al., 2020) clearly decreased the mechanical allodynia and muscle strength loss induced by anastrozole in mice. In addition to their antinociceptive effect dependent on their action on PAR2, both compounds also modulate mast cell activation, which could contribute to alleviating anastrozole-induced painful symptoms (Kelso et al., 2006; Kennedy et al., 2020; Oliveira et al., 2011). Our results agree with previous studies that demonstrated the involvement of PAR2 in painful disorders (Cheng et al., 2011; Kume et al., 2023; Lucena & McDougall, 2020; Muley et al., 2016; Sakamoto et al., 2016).

Additionally, anastrozole did not cause painful symptoms in global PAR2^−/−^ mice or PAR2 Na_v_ 1.8^−/−^ mice, demonstrating that PAR2 exclusively expressed on the sensory neuron is involved in pain associated with aromatase inhibitors. The reciprocal interaction between PAR2 expressed on sensory neurons and tryptase from mast cells sets up a positive feedback loop (Baral et al., 2019; Gupta & Harvima, 2018; Mittal et al., 2019). Thus, the lack of PAR2 prevents the continuous release of inflammatory mediators, thereby reducing the vicious cycle of mast cell activation and peripheral sensitisation, thus leading to pain relief (Gupta & Harvima, 2018; Héron & Dubayle, 2013).

The intraplantar injection of PAR2 agonists enhanced the mechanical sensitivity in wild-type mice, which was attenuated by pre-treatment with PAR2 negative allosteric modulator AZ3451. The local administration of PAR2 agonists did not affect PAR2^−/−^ mice or PAR2 Na_v_ 1.8^−/−^ mice previously treated with anastrozole, confirming the substantial role of PAR2 in anastrozole-induced musculoskeletal pain. As the action of anastrozole is confined to the periphery (Miyajima et al., 2013) and the PAR2 agonists were locally (i.pl) administered, all the painful symptoms induced by anastrozole observed in this study are most likely to derive from peripheral effects.

Our findings also suggest that targeting the mast cell/tryptase/PAR2 axis in aromatase inhibitor-associated pain may be a promising approach to treating such painful complaints. However, given the broad involvement of the mast cell/tryptase/PAR_2_ axis in several physiological and pathological functions (Gupta & Harvima, 2018; Mai et al., 2021), the balance between their beneficial and adverse effects must be considered. The contribution of other algesic mediators released from mast cells (such as additional proteases, histamine and serotonin) and their respective receptors cannot be excluded. Therefore, more studies are necessary to demonstrate their involvement in the painful symptoms, associated with aromatase inhibitors. Furthermore, the absence of changes in non-evoked pain-related behaviours (spontaneous pain) in the anastrozole pain model cannot be excluded and should be evaluated in a long-term treatment protocol, because such behaviours are typically expected to manifest only during more chronic stages of treatment (Tappe-Theodor & Kuner, 2014).

In conclusion, using pharmacological and genetic approaches, we have shown a novel mechanism underlying pain induced by anastrozole, a clinically used aromatase inhibitor. Our data showed that anastrozole mobilised mast cells in peripheral tissue without directly activating PAR2. Mast cell mobilisation in peripheral tissues leads to the release of tryptase, which then activates PAR2, primarily expressed on sensory neurons, contributing to the ongoing painful symptoms associated with anastrozole use. As no PAR2 antagonists have been used clinically until now, mast cell membrane stabilisers, such as ketotifen fumarate, could be assessed in randomised, controlled clinical studies in patients reporting painful symptoms and in treatment with aromatase inhibitors, such as anastrozole, to investigate whether they can alleviate the pain in these patients.

## Supplementary Material

Supplementary Figure 2

Supplementary Figure 3

Supplementary Figure 1

Additional supporting information can be found online in the Supporting Information section at the end of this article.

## Figures and Tables

**FIGURE 1:**
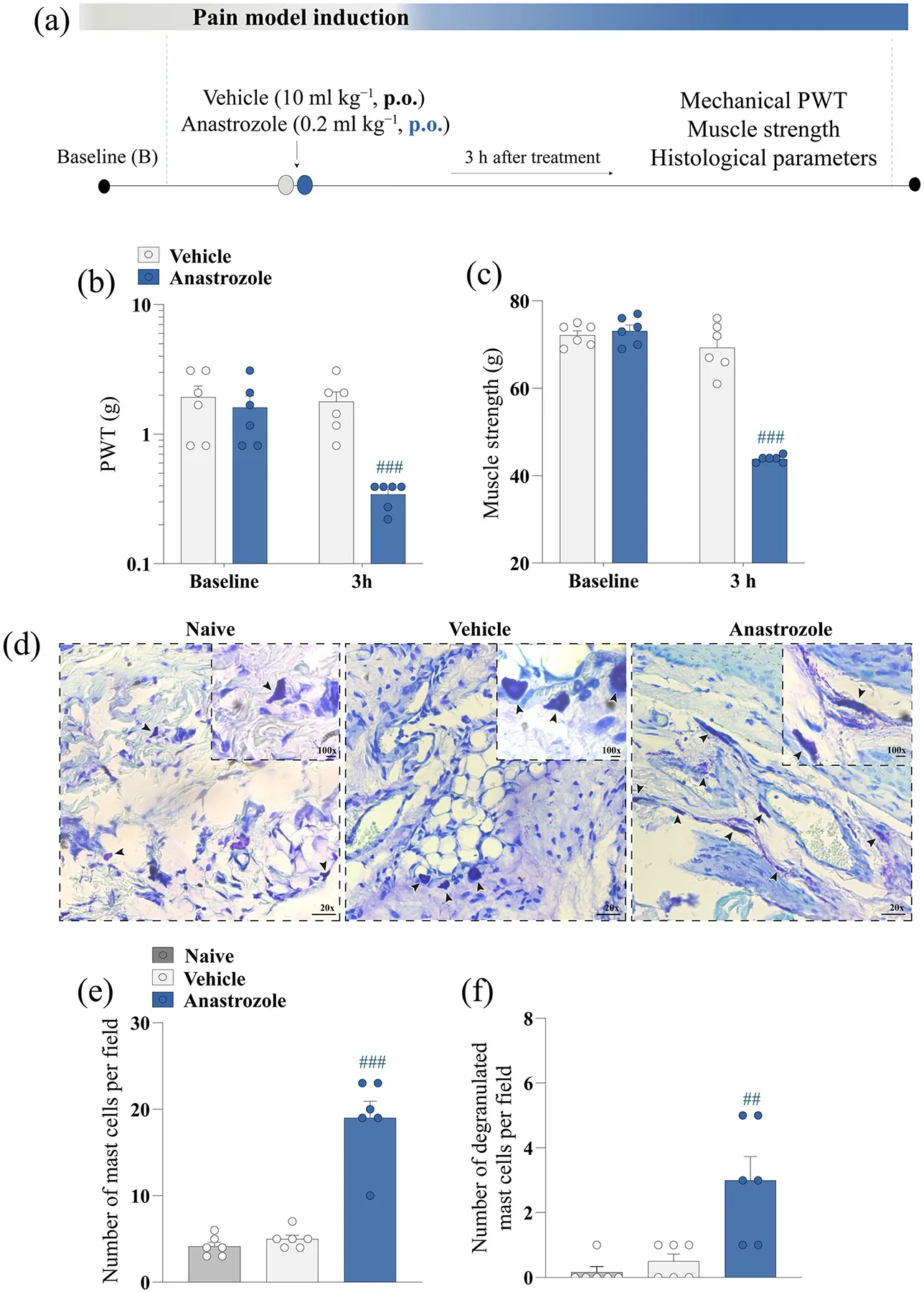
Peripheral mast cells are involved in the anastrozole-induced painful symptoms. (a) Experimental timeline. Oral (p.o.) administration of anastrozole (0.2 mg kg^−1^) in mice reduced (b) mechanical PWT and induced (c) muscle strength loss compared to the vehicle group at 3 h after treatment. (d) Representative photomicrograph of toluidine blue staining and (e) the cumulative number of mast cells per field (objectives at 20X) and (f) the number of degranulated mast cells (objectives at 20X and 100X) in the plantar tissue of mice. Black arrows indicate the presence of mast cells in plantar tissue. Scale bar: 100 μm. Baseline values were measured before anastrozole administration. Data shown are individual values with means + SEM (*n* = 6 per group). ^##^*P* < 0.01 and ^###^*P* < 0.001, significantly different from vehicle group; two-way ANOVA followed by the Bonferroni post hoc test or by one-tailed Student’s *t*-test. PWT: paw withdrawal threshold.

**FIGURE 2:**
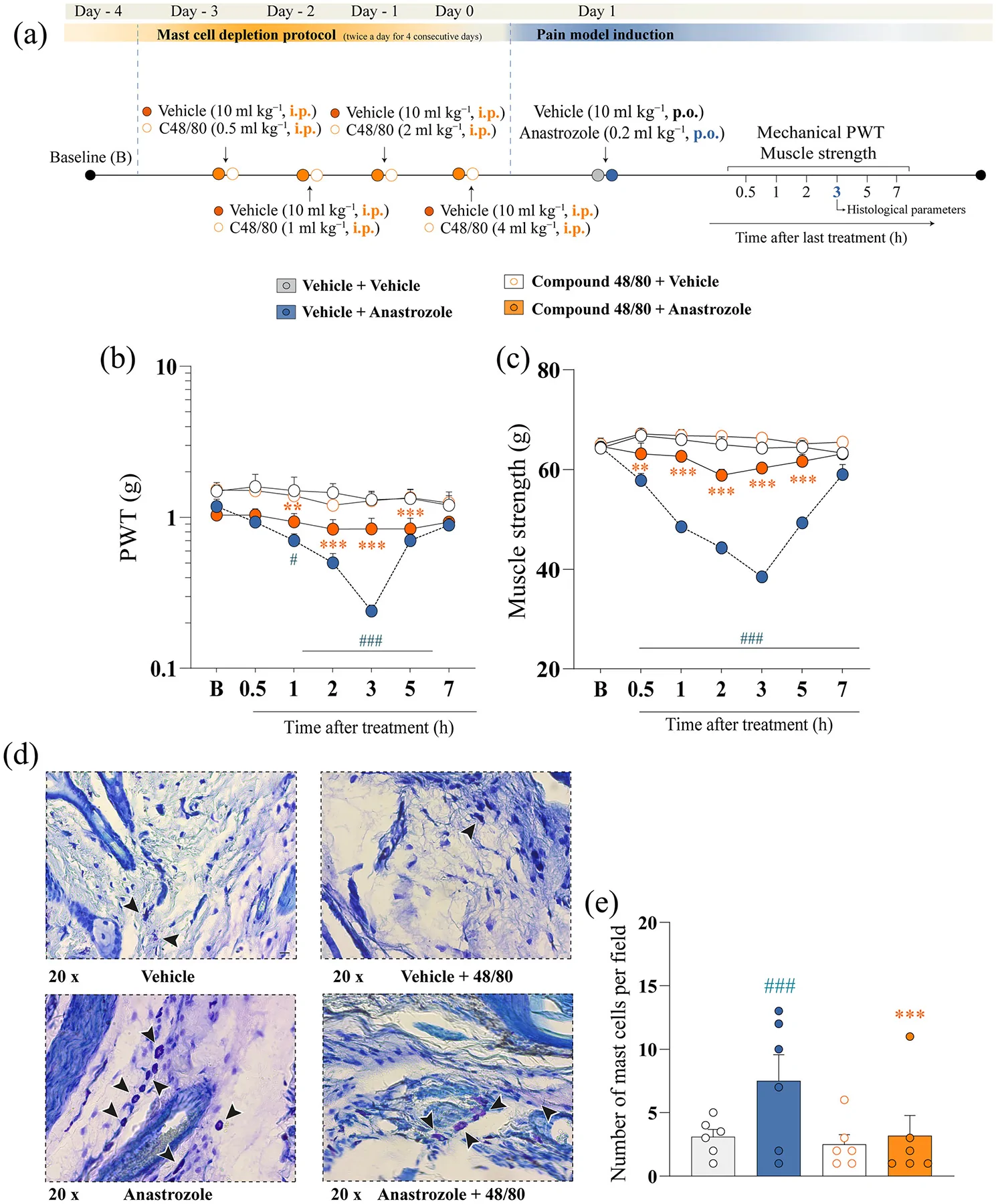
Depletion of mast cells prevents the anastrozole-induced painful symptoms. (a) Experimental timeline. Mice were pre-treated with increasing doses of compound 48/80 (0.5, 1, 2 and 4 mg kg^−1^, i.p.) or its vehicle (10 ml kg^−1^, i.p.) twice a day for four consecutive days to deplete mast cells. On the fifth day, mice were treated (p.o.) with anastrozole (0.2 mg kg^−1^) or its vehicle (10 ml kg^−1^), to induce painful behaviours. (b) Mechanical PWT and (c) muscle strength were assessed before (baseline values) and at different times (from 0.5 to 7 h) after anastrozole or vehicle treatment. (d) Representative photomicrographs (objectives at 20X) of toluidine blue staining and (e) the cumulative number of mast cells/field in the plantar tissue was obtained by counting two fields from three different histological slides, totalling *n* = 6 per group. Black arrows indicate the presence of mast cells in plantar tissue. Scale bar: 100 **μ**m. Baseline (*B*) values were measured before anastrozole administration. Data shown are individual values with the means + SEM (*n* = 6 per group). ^#^*P* < 0.05, ^###^*P* < 0.001, significantly different from vehicle plus vehicle group. **P < 0.01, ***P < 0.001, significantly different from vehicle plus anastrozole group; one- or two-way ANOVA followed by the Bonferroni post hoc test. PWT: paw withdrawal threshold.

**FIGURE 3:**
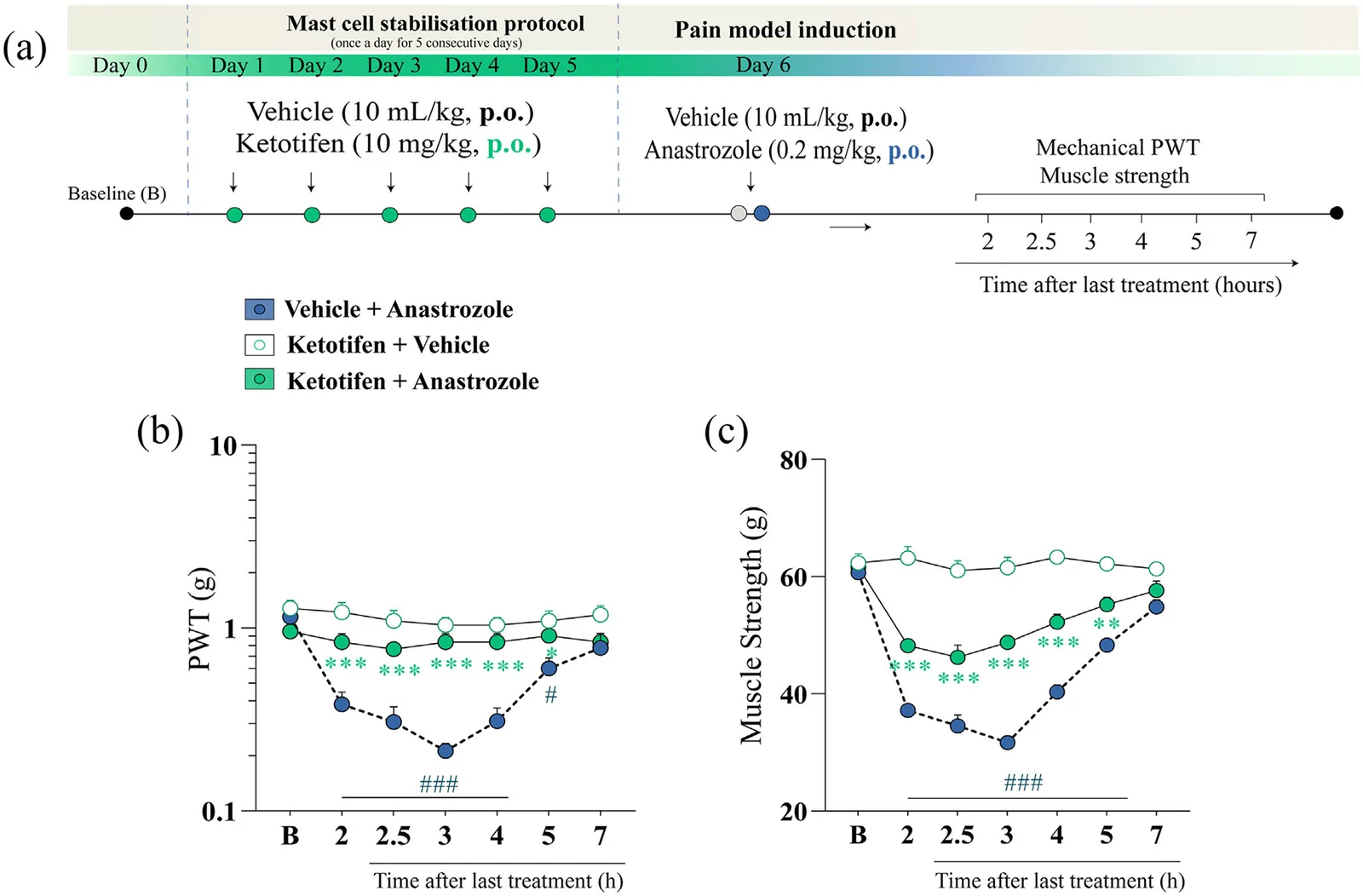
Mast cell membrane stabilisation prevents the painful symptoms induced by anastrozole. (a) Experimental timeline. Mice were pre-treated with ketotifen fumarate (10 mg kg^−1^, p.o.) once daily for five consecutive days to stabilise the mast cell membrane and prevent degranulation. On the sixth day, the mice were treated with anastrozole (0.2 mg kg^−1^, p.o.) or its vehicle (10 ml kg^−1^, p.o.) to induce painful behaviours. (b) Mechanical PWT and (c) muscle strength were assessed before (baseline) and from 2 up to 7 h after anastrozole and vehicle treatment. Basal (*B*) values were measured before anastrozole administration. Data shown are means + SEM (*n* = 6 per group). ^#^*P* < 0.05, ^###^*P* < 0.001, significantly different from *B* values; *P < 0.05, **P < 0.01, ***P < 0.001, significantly different from ketotifen plus anastrozole group; two-way ANOVA followed by the Bonferroni post hoc test. PWT: paw withdrawal threshold

**FIGURE 4:**
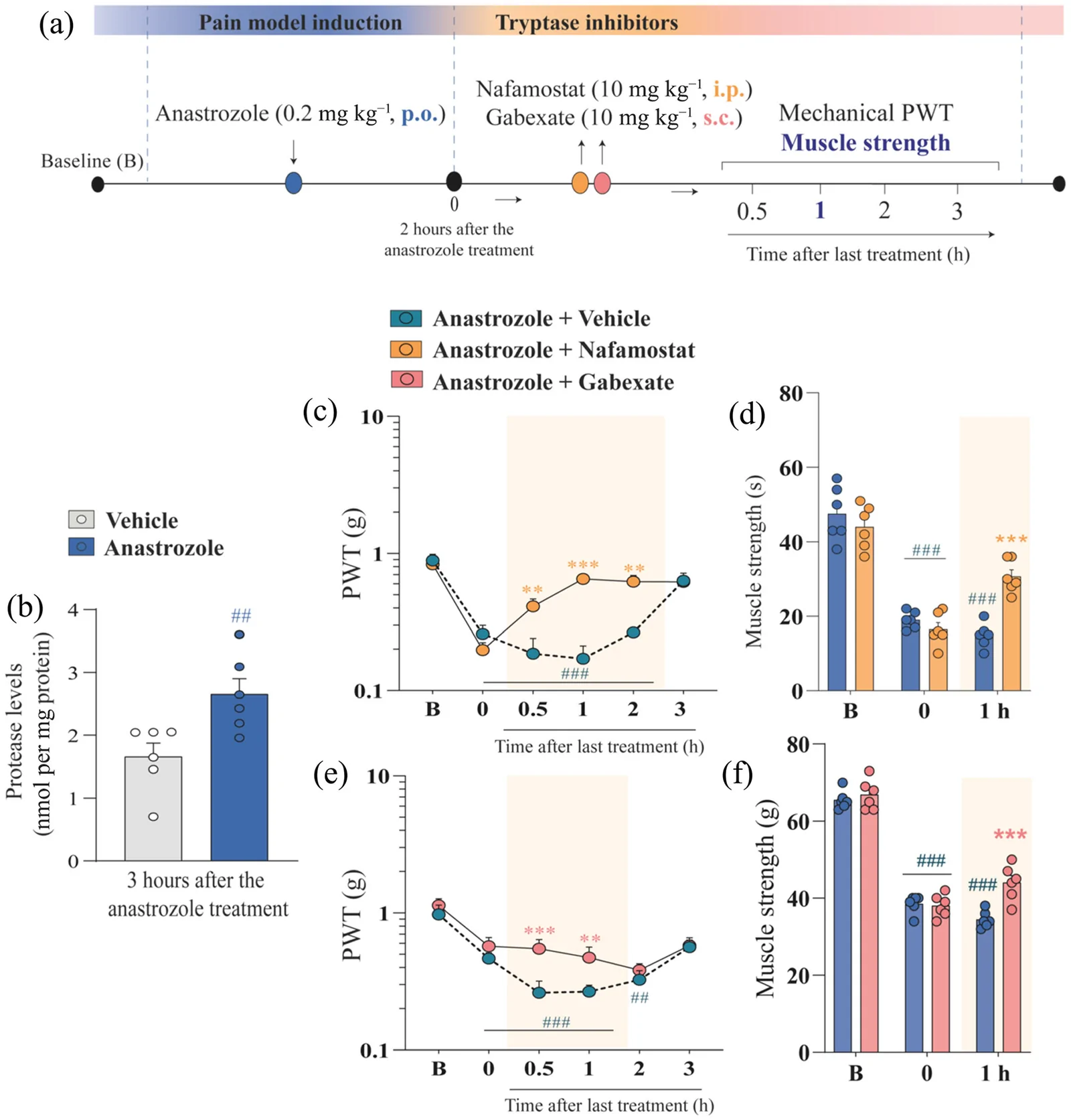
Tryptase contributes to the painful symptoms induced by anastrozole in mice. (a) Experimental timeline. Mice treated with anastrozole (p.o., 0.2 mg kg^−1^) presented (b) increased levels of protease (including tryptase) in the plantar tissue compared to the vehicle group (10 ml kg^−1^, p.o.) at 3 h after treatment. Anastrozole caused mechanical allodynia and muscle strength loss at 2 h after its administration (Time 0), compared with baseline (*B*) values. The effects of two tryptase inhibitors, nafamostat (10 mg kg^−1^; i.p.) or gabexate (10 mg kg^−1^; s.c.) on the changes of (c and e) mechanical PWT and (d and f) muscle strength, induced by anastrozole, was evaluated from 0.5 to 3 h, and at 1 h after treatment with the inhibitor, respectively. Baseline (*B*) values were measured before anastrozole administration. Data shown are the means + SEM, with individual values (d, f); *n* = 6 per group. ^##^*P* < 0.01, ^###^*P* < 0.001, significantly different from *B* values; **P < 0.01, ***P < 0.001, significantly different from anastrozole plus vehicle group; Student’s *t*-test, one-tailed or two-way ANOVA followed by the Bonferroni post hoc test. PWT: paw withdrawal threshold.

**FIGURE 5:**
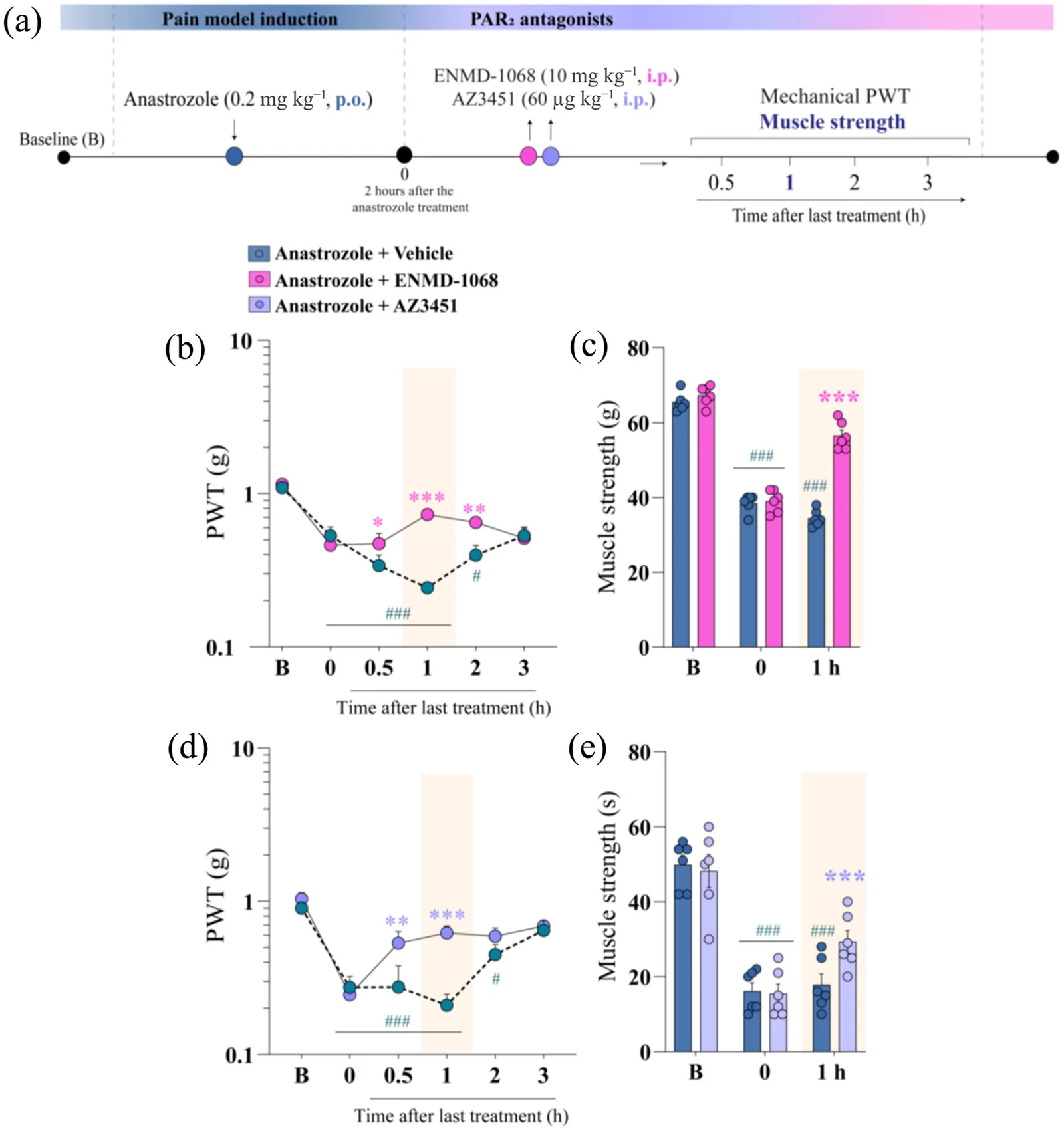
Anastrozole-induced painful behaviours are mediated by PAR2 in mice. (a) Experimental timeline. Mice treated with anastrozole (0.2 mg kg^−1^; p.o.) presented mechanical allodynia and muscle strength loss at 2 h after its administration (Time 0) compared to baseline (*B*) values. The effects of both PAR2 inhibitors, ENMD-1068 (10 mg kg^−1^; i.p.) and AZ3451 (60 μg kg^−1^, i.p.) on the (b and d) mechanical PWT and (c and e) muscle strength were evaluated from 0.5 to 3 h, and at 1 h after treatment with the antagonist, respectively. Baseline (*B*) values were measured before anastrozole administration. Data were expressed as the means + SEM, with individual values (c, e); *n* = 6 per group. ^#^*P* < 0.05, ^###^*P* < 0.001, significantly different from *B* values. *P < 0.05, **P < 0.01, ***P < 0.001, significantly different from anastrozole plus vehicle group. two-way ANOVA followed by the Bonferroni post hoc test. PWT: paw withdrawal threshold.

**FIGURE 6:**
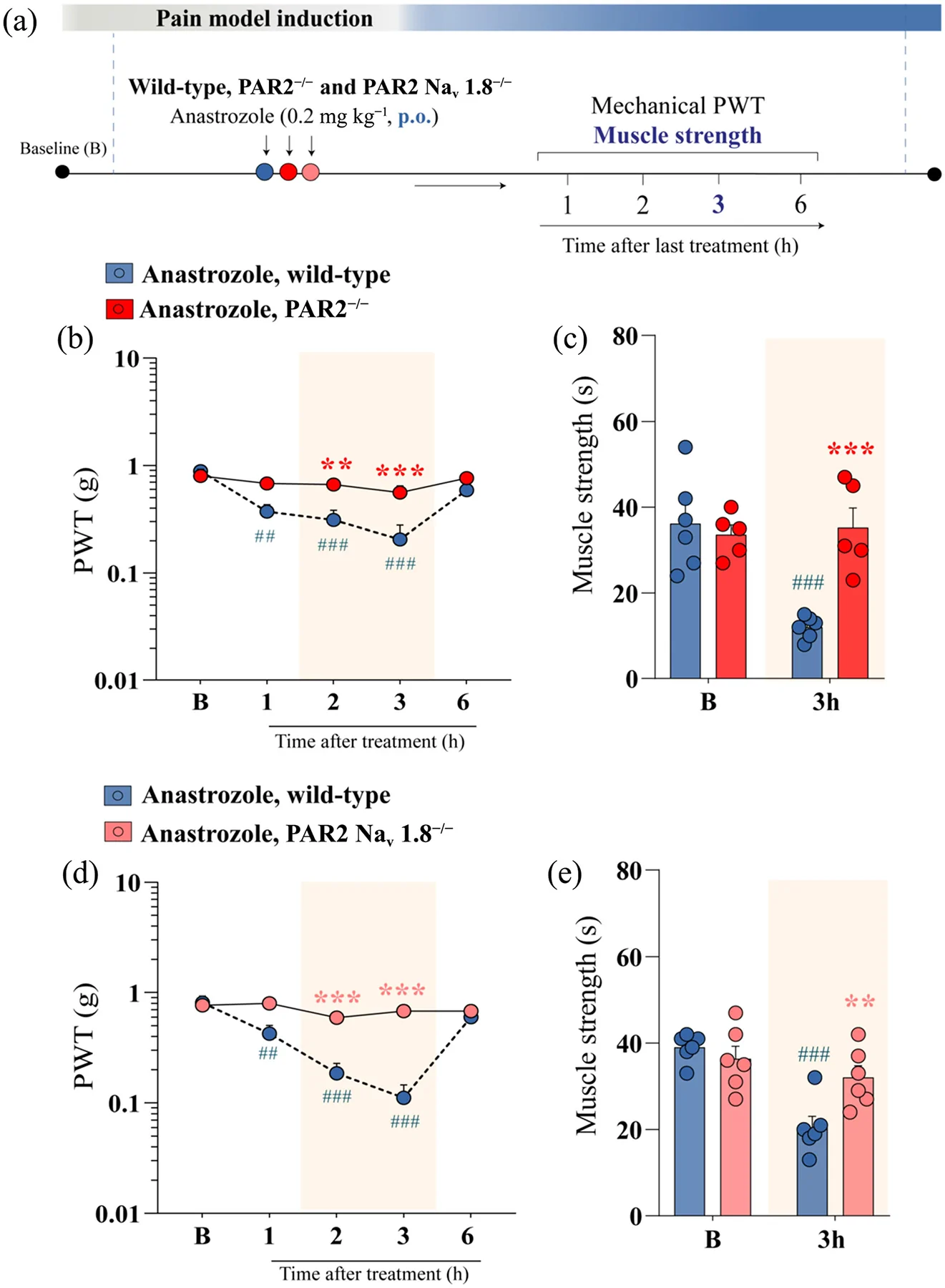
PAR2^−/−^ and PAR2 Nav 1.8 ^−/−^ mice exhibit decreased levels of painful symptoms induced by anastrozole. **(a) Experimental timeline.** Wild-type mice treated with anastrozole (0.2 mg kg^−1^; p.o.) presented mechanical PWT and muscle strength loss, compared with the basal values. The genetic deletion of global (PAR2^−/−^) or sensory neuron-specific PAR_2_ (PAR2 Na_v_ 1.8^−/−^) attenuated the reduction of (b and d) mechanical PWT and (c and e) loss of muscle strength caused by anastrozole. Baseline (*B*) values were measured before anastrozole administration. Data are expressed as the mean + SEM, with individual values (c, e); *n* = 5–6 per group. ^##^*P* < 0.01, ^###^*P* < 0.001, significantly different from *B* values; **P < 0.01, ***P < 0.001 significantly different from anastrozole plus vehicle group wild-type group; two-way ANOVA followed by the Bonferroni post hoc test. PWT: paw withdrawal threshold.

**FIGURE 7:**
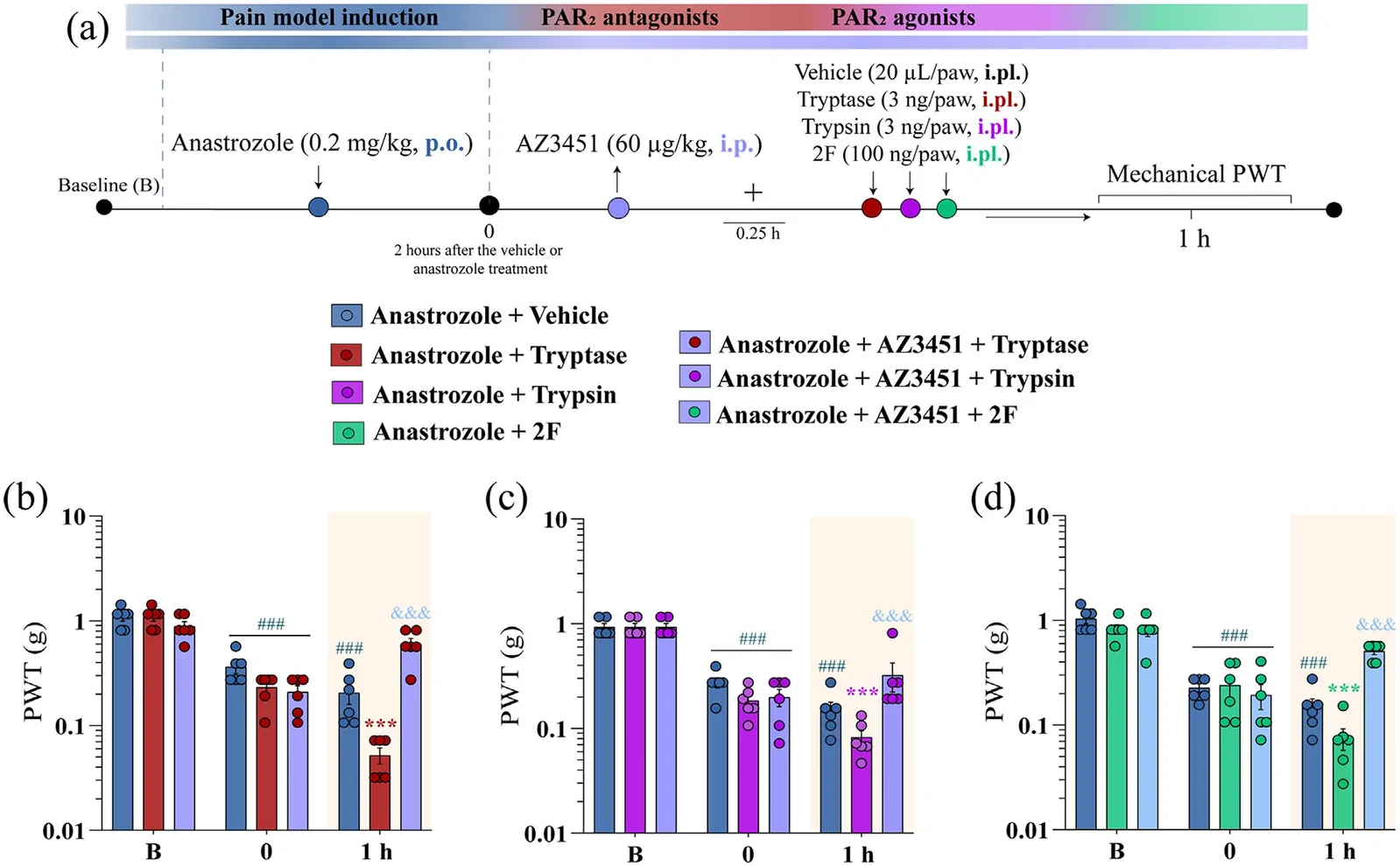
PAR2 agonists (tryptase, trypsin or 2F) enhance the mechanical sensitivity induced by anastrozole. (a) Experimental timeline. Mice were treated with anastrozole (p.o., 0.2 mg kg^−1^) to induce painful behaviours. At 2 h after anastrozole administration (Time 0), vehicle (20 μl/paw), (b) tryptase (3 ng/paw, 20 μl), (c) trypsin (3 ng/paw, 20 μl) or (d) 2F (100 ng/paw, 20 μl) were injected intraplantar (i.pl.). Anastrozole-treated mice received a single treatment of the PAR2 negative allosteric modulator, AZ3451 (60 ug kg^−1^, i.p.), and 0.25 h later, mice received i.pl. injection of tryptase, trypsin or 2F. The mechanical PWT was assessed 1 h after agonist injection. Baseline (*B*) values were measured before anastrozole administration. Data are individual values with the mean + SEM (*n* = 6 per group). ^###^*P* < 0.001, significantly different from *B* values. ***P < 0.001, significantly different from anastrozole plus vehicle group. ^&&&^*P* < 0.001, significantly different from anastrozole plus tryptase, trypsin or 2F group; two-way ANOVA followed by the Bonferroni post hoc test. PWT: paw withdrawal threshold.

**FIGURE 8:**
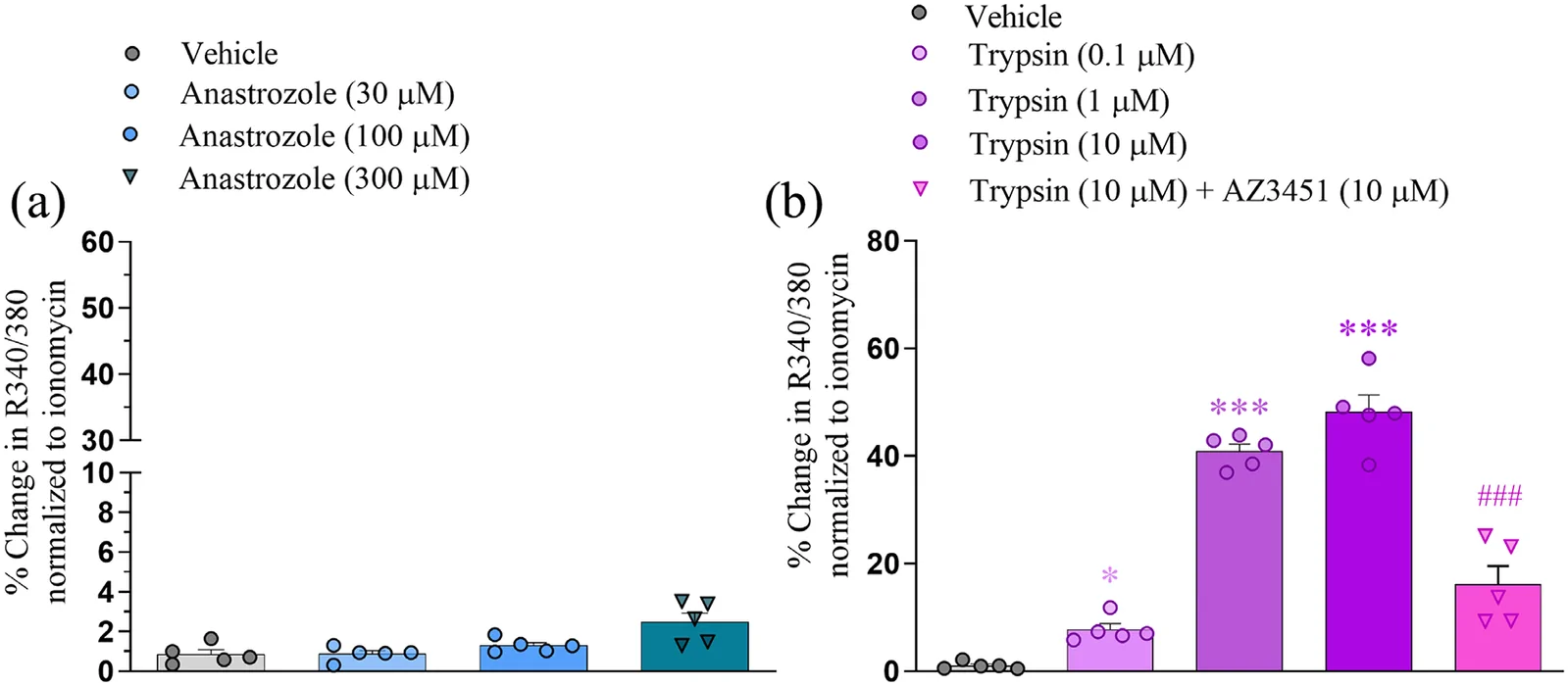
Anastrozole does not directly activate human PAR2. Cumulative data of the calcium responses in HEK293 cells, which express endogenous PAR2 (hPAR2–HEK293) after exposure to vehicle, (a) anastrozole (30–300 μM) or (b) trypsin (0.1–10 μM, PAR2 agonist). PAR2 negative allosteric modulator AZ3451(10 μM) was pre-incubated with trypsin (10 μM). Data are individual values with the means + SEM (*n* = 5 independent experiments). *P < 0.05, ***P < 0.001 significantly different from vehicle group. ^###^*P* < 0.001, significantly different from trypsin (10 μM) group; one-way ANOVA followed by the Bonferroni post hoc test.

## Data Availability

The data that support the findings of this study are available from the corresponding authors upon reasonable request.

## What is already known?

- Aromatase inhibitors improve survival in hormone receptor-positive breast cancer patients.
- Aromatase inhibitors can cause musculoskeletal pain, reducing life quality and treatment compliance in patients.

## What does this study add?

- The mast cell/tryptase/PAR2 axis is essential to the nociception caused by aromatase inhibitors in mice.
- Mast cell degranulation, increased tryptase, and PAR-2 activation contribute to aromatase inhibitor-caused painful symptoms.

## What is the clinical significance?

- The mast cell/tryptase/PAR2 axis represents a therapeutic target in treating aromatase inhibitor-induced painful symptoms.

## Abbreviations:

CMC: carboxymethylcellulose
i.pl.: intraplantar
PAR2 Na_v_ 1.8^−/−^: sensory neuron-specific knockout of PAR2
PAR2: protease-activated receptor 2
PAR2^−/−^: global knockout of PAR2
PWT: mechanical paw withdrawal threshold
2F: 2-Furoyl-LIGRLO-amide
