# Supplementary material for: Critical role of the mast cell/tryptase/PAR2 axis in anastrozole-induced pain
Source: Br J Pharmacol. Author manuscript; Available in PMC 2026 Oct 21. (PMC13684123; doi:10.1111/bph.70280)
Supplement: Supplementary file 1 [file NIHMS2216490-supplement-Supplementary_Figure_3.docx]

**Supplementary Figure 1 - Anastrozole does not cause non-evoked pain behaviour.** **(A)** Experimental timeline. Mice were treated by oral route (p.o.) with anastrozole (0.2 mg/kg) or vehicle (10 mL/kg), and 3 h after the treatments, the non-evoked pain behaviour was recorded for 20 min. **(B)** Ambulation (% activity), **(C)** average velocity of locomotion (centimetres/second), **(D)** track length (centimetres, cm), **(E)** time engaged in grooming (minutes), and **(F)** total activity travelled in the open field (% activity) were recorded. **(G)** Representative images of mice's total activity. Data were expressed as the mean + SEM (n = 6/group) and analysed by Student’s t test, one-tailed.

**Supplementary Figure 2 - PAR_2_ agonists do not alter the anastrozole-induced mechanical PWT in PAR_2_ or PAR_2_ Na_v_1.8** **knockout mice. (A)** Experimental timeline. Wild-type, **(B - D)** global (PAR_2_^-/-^) or **(E - G)** sensory neuron-specific (PAR_2_ Nav 1.8^-/-^) PAR_2_ knockout mice were treated oral route (p.o.) with anastrozole (0.2 mg/kg) to induce painful behaviours. At 2 h after anastrozole administration (Time 0), vehicle (20 µl/paw, intraplantar, i.pl.), **(B and E)** tryptase (3 nmol/paw, 20 µl), **(C and F)** trypsin (3 nmol/paw, 20 µl) or **(D and G)** 2F (100 ng/paw, 20 µl) were injected by the intraplantar route (i.pl.). The mechanical PWT was assessed from 0.5 up to 3 h after the agonists' injection. Basal (B) values were measured before the anastrozole administration. ^#^*p*< 0.01 and ^###^*p*< 0.001 vs. B values. *^**^p*< 0.01 and ^***^*p*< 0.001 vs. anastrozole plus vehicle group (wild-type mice). Data were expressed as the mean + SEM (n=6/group) and analysed by two-way ANOVA followed by the Bonferroni *post hoc* test. PWT: paw withdrawal threshold.
